# Supplementary material for: Immunotherapy for Urological Tumors on YouTubeTM: An Information-Quality Analysis
Source: Vaccines (Basel). 2022 Dec 30;11(1):92. doi: 10.3390/vaccines11010092 (PMC9866846; doi:10.3390/vaccines11010092)
Supplement: Supplementary file 1 [file vaccines-11-00092-s001.zip › vaccines-2071581-supplementary.pdf]

**Table S1.** Search keyword combinations used on YouTube™ on 25 March 2022.

| | **Search Keyword Combinations** |
|---|---|
| 1 | Immunotherapy |
| 2 | Cancer immunotherapy |
| 3 | Renal cancer immunotherapy |
| 4 | Kidney cancer immunotherapy |
| 5 | Bladder cancer immunotherapy |
| 6 | Muscle-invasive bladder cancer immunotherapy |
| 7 | Non-muscle invasive bladder cancer immunotherapy |
| 8 | Urothelial carcinoma immunotherapy |
| 9 | Prostate cancer immunotherapy |
